# Common Adverse Effects of Anti-TNF Agents on Gestation

**DOI:** 10.1155/2016/8648651

**Published:** 2016-12-01

**Authors:** Zacharias Fasoulakis, Panagiotis Antsaklis, Nikolaos Galanopoulos, Emmanuel Kontomanolis

**Affiliations:** ^1^Department of Obstetrics & Gynecology, Democritus University of Thrace, Alexandroupolis, Greece; ^2^1st Department of Obstetrics & Gynecology, University of Athens, Athens, Greece; ^3^State Clinic of Rheumatology, Democritus University of Thrace, Alexandroupolis, Greece

## Abstract

Autoimmune disease has affected up to 50 million Americans, according to the American Autoimmune Related Diseases Association (AARDA) and 75 percent of those affected are women. These inflammatory diseases have variable activity and a lot of women will have to undergo major therapies during and after pregnancy. Many of the women suffering from these disease will improve during gestation. However a lot of women will require continuation of disease-modifying therapies (i.e., biological therapies) throughout pregnancy and post-partum involving many risks. In the past decade all gaze turned to biological therapies, as an attempt, to obtain even more effective medications in order to suppress the exacerbation of autoimmune disease, even at the most unfit circumstances such as pregnancy. The results are both satisfying and promising since increasingly proven thoughts prevail on making anti-TNF agents first-line medications, clearing up the limited knowledge over human influence. The purpose of this review is to summarize the results of the reports with the highest and representative range of patients of the last decade involving the use of anti-TNF agents during pregnancy.

## 1. Introduction

Autoimmune disease affects up to 50 million Americans, according to the American Autoimmune Related Diseases Association (AARDA) and 75 percent of those affected are women. These inflammatory diseases have variable activity and a lot of women will have to undergo major therapies during and after pregnancy. The majority of these women suffering from these diseases will improve during pregnancy. However, a lot of women will require the continual administration of these drugs.

Tumor necrosis factor (TNF) is a substance that is secreted primarily by cells of the immune system and plays a central role in the inflammatory process. It was associated with the pathogenesis of bacterial sepsis when the first preclinical studies using anti-TNF antibodies were performed on animal models (mice) of sepsis [1]. However, it was not until 1991 that studies on a transgenic mouse model of overexpressed human TNF*α* promoted the role of TNF in the development of polyarthritis and that anti-TNF therapies could be effective against the human disease [2].

Today, since therapeutic options are limited during pregnancy, most age- and gender- related incident rates of inflammatory diseases occur during the peak reproductive years and seeing that certain DMARDs are proven to be teratogenic (i.e., methotrexate), studies on other types of immunosuppressive drugs had to be conducted, making anti-TNF drugs the most possible therapy. In the last years more and more data regarding exposure to anti-TNF therapy during pregnancy has been published. Since pregnant patients face a disease both persistent and active, treatment with these agents may be required. Thus, it is crucial to review the available information on the safety of anti-TNF therapies [3, 4].

## 2. Antitumor Necrosis Factor Drugs

Antitumor necrosis factor-*α* (TNF*α*) drugs are a preferable therapeutic option for patients with most autoimmune deceases since they are considered category B drugs by the FDA and they are often well tolerated by patients (even though the inconvenience of intravenous administration (IV), the high costs, and the adverse effects associated with these drugs limit their wide use as first-line medications) [5].

Thanks to modern biotechnology various types of compositions have been produced that selectively inhibit the action of TNF. These compounds include antibodies to TNF (infliximab, adalimumab, certolizumab pegol, and golimumab) or agents that block the TNF receptor (etanercept), that is, the substance with which the TNF needs to be connected to exert its action.

The cytokine inhibitors affecting TNF*α* currently licensed are available [6].

### 2.1. Infliximab (Remicade™, Remsima™, and Inflectra™)

*Infliximab* is a chimeric monoclonal antibody that counters tumor necrosis factor alpha (TNF*α*) and is approved by the US Food and Drug Administration (FDA) as treatment for autoimmune diseases such as inflammatory bowel disease (Crohn's disease and ulcerative colitis), psoriasis, psoriatic arthritis, ankylosing spondylitis, and rheumatoid arthritis [7]. Infliximab does not actively cross the placenta during the first trimester but undergoes efficient placental transfer during the late second and third trimesters and is detectable in the infant's serum for several months after birth [8].

### 2.2. Etanercept (Enbrel™)

Etanercept is a dimeric molecule, an inhibitor acting as a soluble receptor. It binds to TNF*α* and decreases its role in disorders involving excess inflammation in ankylosing spondylitis, adolescent rheumatoid arthritis, psoriasis, psoriatic arthritis, and rheumatoid arthritis and, potentially, in a variety of other disorders mediated by excess TNF*α* [9].

### 2.3. Adalimumab (Humira™, Exemptia™)

Adalimumab is a monoclonal antibody that also targets the TNF alpha (TNF*α*) receptors and it is also used as a treatment for inflammatory bowel disease (Crohn's disease and ulcerative colitis), rheumatoid arthritis, psoriatic arthritis, ankylosing spondylitis, moderate to severe chronic psoriasis, moderate to severe hidradenitis suppurativa, and adolescent idiopathic arthritis. In rheumatoid arthritis, adalimumab has a response rate similar to methotrexate and, in combination, almost doubles the response rate of methotrexate alone [10].

### 2.4. Golimumab (Simponi™)

Golimumab is a human monoclonal antibody that also targets the TNF and it is currently used to treat moderate to severe active psoriatic arthritis, rheumatoid arthritis, and ankylosing spondylitis [11]. In 2013 golimumab also received approval for use on adults with moderate to severe active ulcerative colitis [12].

### 2.5. Certolizumab Pegol (Cimzia™)

Certolizumab pegol is a recombinant, humanized antibody Fab fragment against tumor necrosis factor alpha (TNF*α*), which is expressed in* Escherichia coli* and conjugated with polyethylene glycol (PEG). It is used for the treatment of Crohn's Disease and rheumatoid arthritis [13].

To summarize, three of the anti-TNF agents (namely, infliximab, adalimumab, and golimumab) are structurally complete IgG1 monoclonal antibodies that interact with neonatal Fc receptor (RnFc) and can transfer IgG from mother to fetus through the placenta [14]. Etanercept is a fusion protein directed against the TNF receptor [15], and certolizumab pegol is an incomplete antibody that does not have an Fc part; thus it cannot interact with RnFc [16].

Since these biological agents are either fully chimeric or humanized antibodies, they can cross the placenta at the end of the second and the third trimester.

As mentioned before the United States Food and Drug Administration (FDA) classifies all 5 biologic agents as pregnancy category B drugs; animal reproduction studies have not demonstrated a fetal risk but have no corresponding human studies or animal studies that have shown adverse effects that have not been confirmed in controlled studies of women in the first trimester (or subsequent trimesters) [12].

## 3. Anti-TNF Agents: Crucial Studies

As mentioned, since the highest age- and gender-related incidence rates of inflammatory bowel disease (IBD; Crohn's disease, ulcerative colitis) occur during the peak reproductive years [3], a lot of important questions have been raised concerning the safety of use for both mother and fetus. Thus, over the past decade, a number of studies were conducted in order to provide answers considering the use of anti-TNF agents during pregnancy (Table 1).

In 2004 Katz et al. published in The American Journal of Gastroenterology a study of 131 women administered infliximab during pregnancy. The results were unequivocal. Of the 146 identified pregnancies, 131 involved women exposed directly to infliximab and resulting data was available for 96 of these women. With miscarriages in only 14 of the 96 pregnancies (15%), therapeutic termination in 18 out of 96 (19%), and live births in 64 out of 96 (67%), the authors stated that the results of pregnant women in the US population exposed to infliximab during pregnancy do not differ from pregnant women with CD not exposed to infliximab and also that no increased risk of adverse outcome was detected but in order to exclude any fetal risk, follow-up of larger numbers of pregnant women exposed to infliximab proved to be necessary [17].

In the same year (2004) Chambers et al. presented data of the Organisation of Teratology Information Services of 33 women in gestation (divided in 2 groups depending on the treatment administered). 29 out of 33 pregnant were treated with etanercept while 4 were on infliximab, all of whom were in their first trimester. Even though out of the 29 pregnancies exposed to etanercept one ended in trisomy 18 and 3 in spontaneous abortion, the authors concluded that no significant differences in the rate of malformation were observed between those two groups [18].

It was in 2005 that Mahadevan et al. conducted the first study on intentional use of infliximab during gestation to maintain remission of Crohn's disease. In this study 10 participants received infliximab infusions for induction and maintenance of remission of Crohn's disease during pregnancy (8 treated for remission, 1 started therapy at 3rd trimester, and 1 during 1st trimester). The results were 4 women exhibiting no symptoms (from conception to labor), 2 women showing improvement of their symptoms, and 2 patients that relapsed. Furthermore, those 10 women gave birth to live fetuses without congenital or other abnormalities associated with infliximab [19].

In 2005 Joven et al. presented, among women exposed to biological therapies in rheumatic diseases, 13 women (14 pregnancies) treated with anti-TNF*α* agents (4 with infliximab, 8 with etanercept, and 2 with adalimumab). All pregnancies resulted in expected range with no miscarriages or other complications associated with the use of the anti-TNF drugs [20].

In 2007 Strangfeld et al. analyzed the data of 545 patients treated with biologic agents either during pregnancy or before conception, from a prospective cohort study, involving patients with rheumatoid arthritis, conducted by the German biologics register (RABBIT). The authors compared data from patients who were exposed to biologic agents during pregnancy to those who stopped either biologic or conventional DMARD treatment before conception. 37 pregnancies in 29 women could be analyzed. Anti-TNF agents were given to 27 of these pregnant women before conception (2 patients were treated with infliximab, 20 with etanercept, and 5 with adalimumab). The authors did not find a relation between the use of anti-TNF agents prior to conception and miscarriages, higher congenital malformations, or low birth weight [21].

The British Society for Rheumatology Biologics Register in 2011 published a review by Verstappen et al. of 130 pregnancies concerning patients receiving anti-TNF agents before or during pregnancy. All data was collected and summarized with regard to adverse effects including pregnancies with rheumatoid arthritis patients treated with anti-TNF [22]. The patients were divided in groups according to the use of anti-TNF combined with methotrexate and/or leflunomide or used as monotherapy at the time of conception or prior to it (73 patients treated with etanercept, 30 treated with infliximab, and 26 treated with adalimumab). Patients exposed to anti-TNF*α* therapy at the time of conception had a higher spontaneous abortion rate in contrast to patients without prior exposure (17%) and those in the control group (10%). Thus, the authors concluded that even though the results are promising it is preferable to avoid anti-TNF therapy at the time of conception [22].

Schnitzler et al. recorded the pregnancy results of 212 women in 2011 with inflammatory bowel disease under infliximab and adalimumab treatment. Forty-two pregnancies exposed directly to anti-TNF agents (35 with infliximab and 7 with adalimumab) were compared to 23 pregnancies before inflammatory bowel disease diagnosis, 78 pregnancies before starting infliximab, 53 pregnancies treated with infliximab, and 56 pregnancies of healthy women. Thirty-two of 42 pregnancies resulted in live fetuses. Seven premature deliveries occurred, 6 children with low weight, and one stillbirth. One boy weighed 1630 g delivered in 33rd week and died of necrotic colitis 13 days later. Eight miscarriages were observed and one trisomy 18 on a 37-year-old pregnant woman was terminated. The authors stated that direct exposure to anti-TNF*α* therapy did not result in increased adverse pregnancy outcomes, but it was worse, compared to pregnancies before inflammatory bowel disease diagnosis [23].

Mahadevan et al. published another study considering the placental transfer of anti-TNF agents with IBD in 2013. 31 pregnant women were treated with infliximab (11), adalimumab (10), and certolizumab pegol (10). The median level of infliximab and adalimumab was measured 160% and 153% in the cord compared to that of the mother. In contrast the median ratio of cord to maternal certolizumab pegol level was 3.9%. Even though the authors concluded that certolizumab pegol has a very low placental transfer, no statistically important result could be derived since there were no birth defects or serious adverse outcomes related to biological agents used in any of the gestations [24].

The Journal of Rheumatology published a MEB Clowse et al.'s study in 2015 of maternal exposure to certolizumab pegol on the 1st trimester; a third out of them continued the drug into the second and/or third trimesters, in 339 pregnancies, and paternal exposure in 33 pregnancies (372 known outcomes out of 625 pregnancies since September 2014). In maternal exposure pregnancy the outcome was categorized into live births (*n* = 254), miscarriages (*n* = 52 fetal death before 20th week of gestation), induced abortion (*n* = 32), and stillbirth (*n* = 1 fetal death after 20th week of gestation). The authors stated lack of harmful effects of certolizumab pegol exposure during gestation; however more experimentation is needed to confirm such a claim [25].

More recently, Hoxha et al. (2016) presented a study of 38 pregnant women out of whom 24 were exposed to anti-TNF agents at conception/1st trimester, 11 were exposed prior to conception, and 3 followed paternal exposure. Of the 38 pregnancies, one infant exposed to adalimumab at conception/1st trimester was diagnosed with congenital diaphragmatic hernia and obstructive megaureter and one fetus exposed to etanercept 4 weeks before conception was diagnosed with trisomy 16. Study results suggested that anti-TNF agents could be safe when administered during conception/I trimester [26].

In 2016 also, the Korean Association for the Study of Intestinal Diseases published a cross-sectional study by Komoto et al., of the outcomes of exposure to biological agents and thiopurines in pregnant women. Disease, age, duration of disease, smoking, and drinking were characteristics taken into consideration. The patients were divided in 4 groups according to use of biologic agents only (*n* = 24), biologic agents combined with thiopurine (*n* = 10), thiopurine only (*n* = 7), or neither biological agents nor thiopurine exposure (*n* = 31). Out of these patients 23 were treated with infliximab, 1 with adalimumab, and 10 with both infliximab and thiopurines. Between the groups no differences concerning low birth weight, premature birth, birth weight, or congenital abnormalities were observed. A statistically significant difference however was observed in spontaneous abortion rates to those exposed to anti-TNF agent group [27].

Information concerning the use of these agents has also been derived by case reports throughout these years.

Three case reports of adalimumab use during pregnancy support its administration during gestation even though there is a lack of studies on the use of adalimumab on humans. Three women with chronic or active Crohn's disease used adalimumab during gestation. The first study concerned a 34-year-old woman with active disease during conception. The patient received 38 doses of 40 mg during gestation [28]. The second presented the case of a 34-year-old woman with a persistent disease that had previously undergone proctocolectomy with ileoanal pouch for presumed ulcerative colitis 7 years prior to presentation who [29], due to persisting disease (initially placed on high dose prednisone 60 mg/d), started azathioprine 100 mg/d and adalimumab 80 mg initially and 40 mg every other week. The third study involves a 35-year-old woman that underwent subtotal colectomy and ileorectal anastomosis due to persistent Crohn's disease and became pregnant 7 months after starting therapy with adalimumab. By her own decision she continued therapy with adalimumab. She was admitted in the hospital for abdominal pain and fever in her 20th week and improved after therapy with steroids for 2 weeks [30]. All three women gave birth to healthy infants.

Parallel to the above, the European League Against Rheumatism (EULAR) constantly research the use of anti-TNF agents. According to EULAR “continuation of tumor necrosis factor-*α* (TNF*α*) inhibitors during the first part of pregnancy should be considered. Etanercept and certolizumab may be considered for use throughout pregnancy due to the low rate of transplacental passage,” but the need of a better understanding of anti-TNF use with more studies is necessary since the majority take place in the 1st trimester [31].

Even though certolizumab pegol and golimumab are drugs which have been released in recent years, certolizumab is an anti-TNF agent specific for humans; thus there are not enough information since there are no studies on laboratory animals. Also golimumab lack on recorded data of use on human pregnancies. At present, clinical evidence for both golimumab and certolizumab pegol mentioned before does not indicate connection with increased rates of congenital malformations and even certolizumab is considered safe throughout the entire pregnancy period. Due to limited results on golimumab, alternative medications should be considered for treatment during pregnancy [32, 33].

## 4. Conclusion

In the last years the use of biologic therapies has rapidly expanded. They promote an “ally” to disease-modifying antirheumatic drugs (DMARDs) for the treatment of autoimmune and rheumatologic diseases and are considered a big innovation due to the good efficacy and safety profiles (as studies have proven so far), as well as to the better understanding of the initial targets of altered immune regulation and activity in various diseases.

Today, more and more documented case series and reports, registries, and other multicenter cohort studies have been published considering the safety of biological drug use during gestation, gathering important information, and data regarding their use as first-line medical treatments and also the safety for the mother and the fetus during and after gestation. It is now certain that anti-TNF agents can cross the placenta (in the late second and third trimester) raising concerns about fetal growth. By far some of the biological factors have been investigated more while others have been used less in clinical research. Nevertheless, until now research seems promising for the future of these therapies. To date, there is no evidence that anti-TNF agents are associated with teratogenicity, increased pregnancy loss, or embryo toxicity. Even though no firm conclusions can yet be gathered and it is firmly believed that more studies are required to reach safe conclusions, the results are more and more reassuring about the safety of this medications. As human knowledge is limited, therapists should start considering some of these drugs as a therapy of choice more often, balancing the potential risks associating it against DMARDS and steroids, especially when increasingly more data support no increased risk of adverse outcome [17, 24, 31] (Table 2).

## Figures and Tables

**Table 1:** Summary of pregnancy outcomes in studies of women with rheumatic diseases exposed to anti-TNF therapy during pregnancy.

Registry	Pregnancies exposed (maternal exp.)	Disease	Anti-TNF agent administered	Results
Katz et al. 2004 [17]	131 (96 available data)	CD, UC, RA	INF (96 patients)	Live births 64 Miscarriages 14 Theur. Term. 18

Chambers et al. 2004 [18] OTIS	33	RA, CD	INF (4 patients) ETA (29 patients)	Live births ETA: 7 IFX: 2 Miscarriages ETA: 3 IFX: 1 Theur. Term. ETA: 1 IFX: 1 Trisomy 18 ETA: 1

Mahadevan et al. 2005 [19]	10	CD	INF (10 patients)	Live births 10 Premature births 3 Low birth weight 1

Joven et al. 2005 [20]	14	RA, PsA, JIA	INF (4 patients) ETA (8 patients) ADA (2 patients)	Live births 7 Miscarriages 1 Theur. Term. 4 On-going pregnancies 2

Strangfeld et al. 2007 [21] RABBIT	37	RA	INF (2 patients) ETA (20 patients) ADA (5 patients)	No relation between the use of anti TNF agents prior to conception and miscarriages, higher congenital malformations, or low birth weight (4.5% versus 6.6%)

Verstappen et al. 2011 [22] BSRBR	130	RA, PsA, JIA, AS, SLE, AOSD	INF (30 patients) ETA (73 patients) ADA (26 patients)	Live births 88 Theur. Term. 10 Spontaneous abortion was highest among patients exposed to anti-TNF at the time of conception (17% versus 10%)

Schnitzler et al. 2011 [23]	42	IBD	INF (35 patients) ADA (7 patients)	Live births 32 Low birth weight 6 Premature births 3 On-going pregnancies 1

Clowse et al. 2015 [25]	339	RA, CD	CZP (339 patients)	Live births 254 Miscarriages 52 Induced abortion 32 Stillbirth 1

Hoxha et al. 2016 [26]	35	RA, PsA, AS	INF (3 patients) ETA (25 patients) ADA (5 patients) CZP (2 patients)	Live births 31 Spontaneous abortion 4 Premature birth 2 Trisomy 16 1 Congenital diaphragmatic hernia and obstructive megaureter 1

Komoto et al. 2016 [27]	34	IBD	INF (33 patients) ADA (1 patient)	Live births 34 Spontaneous abortion 6 Premature birth 2 Low birth weight 6 Congenital anomaly 1

INF: infliximab; ETA: etanercept; ADA: adalimumab; CZP: certolizumab pegol; Theur. Term.: therapeutic termination; CD: Crohn's disease; RA: rheumatoid arthritis; PsA: psoriatic arthritis; JIA: juvenile idiopathic arthritis; AS: ankylosing spondylitis; SLE: systemic lupus erythematosus; IBD: inflammatory bowel disease; maternal exp.: maternal exposure.

**Table 2:** Summary of studies and results of pregnancy exposure related to anti-TNF agents used to treat rheumatic diseases, SLR-period 2008–2015 [31].

Anti-TNF agent	Comments on miscarriages and/or congenital malformations compared with control groups (live births)	Strength of evidence according to GRADE Oxford
Adalimumab	No significant difference on miscarriages. Increased rate of congenital malformation in one study.	+++ 2b
Certolizumab	No increased rate of miscarriages or congenital malformation. (No studies with control group available.)	++ 3b
Etanercept	No difference on miscarriages or congenital malformation.	+++ 2b
Golimumab	Combined to methotrexate high rate of miscarriages. No indications of increased congenital malformations. (No studies with control group available.)	+ 4
Infliximab	No difference on miscarriages and/or congenital malformation compared to control groups.	++++ 2b
